# Occupational Exposure to Cooking-Generated Polycyclic Aromatic Hydrocarbons and Associated Oxidative Stress and DNA Damage Among Grill Restaurant Workers

**DOI:** 10.3390/toxics14060512

**Published:** 2026-06-12

**Authors:** Sumed Yadoung, Peerapong Jeeno, Phannika Tongchai, Sakaewan Ounjaijean, Kongsak Boonyapranai, Saweang Kawichai, Hataichanok Chuljerm, Kanokwan Kulprachakarn, Anurak Wongta, Surat Hongsibsong

**Affiliations:** 1 School of Health Sciences Research, Research Institute for Health Sciences, Chiang Mai University, Chiang Mai 50200, Thailand; sumed.yadoung@cmu.ac.th (S.Y.); peerapong_jeen@cmu.ac.th (P.J.); phannika.t@cmu.ac.th (P.T.); sakaewan.o@cmu.ac.th (S.O.); kongsak.b@cmu.ac.th (K.B.); hataichanok.ch@cmu.ac.th (H.C.); kanokwan.kul@cmu.ac.th (K.K.); anurak.wongta@cmu.ac.th (A.W.); 2 Environmental & Occupational Health Sciences and Non-Communicable Diseases Research Center, Research Institute for Health Sciences, Chiang Mai University, Chiang Mai 50200, Thailand; sawaeng.kaw@cmu.ac.th

**Keywords:** polycyclic aromatic hydrocarbons, 1-hydroxypyrene, cardiovascular strain, occupational biomonitoring, cooking fume exposure

## Abstract

Street-food grilling is a common occupation in Asia, yet the occupational health risks associated with cooking-generated polycyclic aromatic hydrocarbons (PAHs) exposure, occurring alongside plausible unmeasured co-exposures such as ambient heat and physical workload, remain under-researched. This study investigated the internal dose of PAH exposure and its association with early biological effects and physiological strain among grill restaurant workers. A cross-sectional study was conducted involving grill workers and 20 age/BMI-matched controls. Urinary 1-hydroxypyrene (1-OHP) was utilized as the primary exposure biomarker. The study assessed early biological effects such as oxidative stress (8-OHdG, F_2_-isoprostanes), lung epithelial integrity (CC16), and genotoxicity (BPDE-DNA adducts) via ELISA. Physiological parameters, including blood pressure and heart rate, were recorded to evaluate acute cardiovascular strain. Workers had significantly elevated urinary 1-OHP levels compared to controls (Hodges–Lehmann ratio = 3.66, 95% CI: 1.68–7.12, representing a 3.7-fold median increase), with exposure levels increasing proportionally to smoke proximity. Notably, workers demonstrated a significantly higher median resting heart rate (HL ratio = 1.13, 95% CI: 1.05–1.23; +12.9%) and systolic blood pressure (HL ratio = 1.09, 95% CI: 1.00–1.18; +8.9%) compared to their office-based peers. Although strong correlations were observed among biological effect biomarkers (*r_s_* = 0.42–0.63), there were no significant differences between groups for 8-OHdG, CC16, or BPDE-DNA adducts, suggesting that cardiovascular parameters reflect acute short-term responses, while genomic damage markers may require higher cumulative exposure thresholds to become detectable. The study revealed that grill restaurant workers face substantial internal PAH exposure and significant cardiovascular strain, occurring alongside plausible unmeasured co-exposures including ambient heat and physical workload. The prevalence of chronic cough and elevated heart rate is a critical early warning sign for occupational health. Our findings indicate that current general ventilation is inadequate, highlighting an urgent need for localized engineering controls and comprehensive health surveillance, including cardiovascular monitoring in the service sector.

## 1. Introduction

Polycyclic aromatic hydrocarbons (PAHs) are a large class of organic compounds generated primarily during incomplete combustion of organic materials such as coal, petroleum products, biomass, and food [1,2]. These compounds are widely distributed in the environment and represent an important group of environmental and occupational pollutants [3]. Human exposure to PAHs can occur through inhalation of contaminated air, ingestion of contaminated food, and dermal contact with polluted particles [4,5]. Several PAHs possess mutagenic and carcinogenic properties, and compounds such as benzo[a]pyrene have been classified as carcinogenic to humans by the International Agency for Research on Cancer (IARC) [6,7]. Epidemiological and toxicological studies have shown that chronic exposure to PAHs may contribute to a variety of adverse health effects, including respiratory diseases, oxidative stress, DNA damage, and an increased risk of cancer [8,9]. Consequently, the assessment of occupational exposure to PAHs and their early biological effects has become an important topic in occupational and environmental health research [4,10].

Cooking activities, particularly grilling, roasting, and frying at high temperatures, have been recognized as important sources of airborne PAHs and other combustion-related pollutants [11,12,13]. During grilling processes, fat and juices from meat can drip onto hot surfaces or charcoal, producing smoke that contains complex mixtures of particulate matter, aldehydes, volatile organic compounds, and PAHs [14,15]. Workers in grill restaurants are therefore likely to experience repeated exposure to cooking fumes during daily work activities, especially in workplaces with inadequate ventilation systems [16,17]. Previous studies have reported elevated concentrations of PAHs in indoor air during cooking activities and increased internal exposure among professional cooks and restaurant workers [14,16,17].

Recent studies have also shown that occupational exposure to cooking emissions can result in measurable increases in biomarkers of PAH exposure among restaurant workers [13,15]. For example, a biomonitoring study of grill restaurant workers reported a significant increase in urinary monohydroxylated PAH metabolites during working periods compared with non-working periods, suggesting substantial occupational exposure associated with grilling activities [17]. Similarly, another study investigating kitchen workers found higher concentrations of urinary PAH metabolites among exposed workers compared to control groups, highlighting the potential occupational health risks associated with cooking fumes [12,16]. Recent reviews have also emphasized that high-temperature cooking methods, particularly grilling and frying, generate substantial amounts of PAHs and may pose long-term health risks for workers in commercial kitchens [3,11]. These findings suggest that workers in grill restaurants may represent a vulnerable occupational group requiring further investigation [17].

Biomonitoring has been widely used to evaluate human exposure to PAHs and to assess potential health risks in exposed populations [1,3]. Urinary metabolites of PAHs are commonly used biomarkers because they reflect internal exposure following inhalation or ingestion of PAHs [17]. Among these metabolites, 1-hydroxypyrene (1-OHP), a metabolite of pyrene, is one of the most widely accepted biomarkers for assessing recent exposure to PAHs in occupational and environmental studies [16,17]. Urinary 1-OHP levels have been shown to correlate with airborne PAH concentrations in several occupational settings, including coke oven workers, asphalt workers, and individuals exposed to cooking emissions [8,10,14]. Recent advances in biomonitoring techniques have further supported the use of urinary PAH metabolites as reliable indicators for evaluating occupational exposure to combustion-related pollutants [3,17].

In addition to exposure assessment, biomarkers reflecting early biological responses are increasingly applied in occupational health research to better understand the mechanisms linking chemical exposure to potential health outcomes [4,7]. Oxidative stress is considered to be one of the major mechanisms underlying PAH toxicity [9,18]. During metabolic activation, PAHs may generate reactive oxygen species that can induce oxidative damage to cellular macromolecules, including DNA, proteins, and lipids [13,15]. Urinary 8-hydroxy-2′-deoxyguanosine (8-OHdG) is widely used as a biomarker of oxidative DNA damage and has been applied in numerous environmental and occupational studies to investigate exposure to air pollutants and combustion products [10,12,16]. In addition, F_2_-isoprostanes are considered to be reliable indicators of lipid peroxidation and systemic oxidative stress and are considered to be one of the most robust biomarkers of oxidative injury in humans [12,18].

Another important pathway of PAH toxicity involves genotoxic damage [6,7]. Certain PAHs, such as benzo[a]pyrene, undergo metabolic activation to highly reactive intermediates, including benzo[a]pyrene diol epoxide (BPDE) [9,10]. These metabolites can bind covalently to DNA and form BPDE–DNA adducts, which may lead to mutations and carcinogenesis if DNA repair mechanisms fail [6,7,19]. Measurement of PAH-DNA adducts has therefore been widely used as a biomarker of early genotoxic effects associated with PAH exposure in occupational and environmental studies [8,20].

In addition to systemic effects, inhalation of airborne pollutants may directly affect the respiratory epithelium. Club cell secretory protein 16 (CC16), a protein produced by club cells in the airway epithelium, has been proposed as a sensitive biomarker reflecting lung epithelial integrity and airway injury. Changes in circulating CC16 concentrations have been reported following exposure to air pollutants and occupational inhalation hazards, suggesting that this biomarker may provide useful information regarding early respiratory effects associated with inhaled toxicants [3].

Although numerous studies have investigated PAH exposure in occupational environments such as coke ovens, asphalt industries, and traffic-related occupations, relatively limited research has focused on restaurant workers who are exposed to cooking-generated pollutants [4,8]. In many Asian countries, grill-style restaurants are widely popular, and workers in these environments may be exposed to cooking fumes for extended periods during daily work [11,17]. Moreover, most previous studies have focused primarily on exposure assessment or environmental monitoring, while relatively few studies evaluated multiple biomarkers representing different stages along the exposure–effect pathway, including exposure biomarkers, oxidative stress markers, lung injury biomarkers, and genotoxic indicators [3,7].

Therefore, the present study aimed to investigate the occupational exposure to PAHs among grill restaurant workers using urinary 1-hydroxypyrene as a biomarker of internal exposure. In addition, biomarkers of oxidative stress (8-OHdG and F_2_-isoprostanes), lung epithelial injury (CC16), and genotoxic damage (BPDE–DNA adducts) were measured to evaluate the potential biological effects associated with PAH exposure. By integrating multiple biomarkers representing different biological mechanisms along the exposure–effect pathway, this study provides a comprehensive assessment of potential occupational health risks among workers exposed to cooking-generated PAHs.

## 2. Materials and Methods

### 2.1. Study Design and Participants

A cross-sectional study was conducted among grill restaurant workers and a control group in Northern Thailand. A total of 58 individuals (38 workers and 20 controls) were included. Grill restaurant workers were recruited from small-to-medium commercial grilling establishments in Chiang Mai. The 20 healthy controls were recruited from sedentary, office-based administrative positions at Chiang Mai University, with no professional contact with commercial cooking fumes, industrial combustion, or occupational chemical hazards. Both cohorts resided and worked within the same urban municipality of Chiang Mai, ensuring a shared baseline exposure to regional ambient air pollution and traffic-related emissions, thereby reducing, though not eliminating, baseline differences in ambient PAH exposure between groups, and allowing the commercial kitchen environment to be considered as the primary differential exposure variable, while acknowledging that residual confounding cannot be fully excluded. Participants were matched by age and BMI. Additional confounder screening was conducted via structured face-to-face questionnaire interviews to exclude individuals with chronic respiratory disease, active medication or dietary supplement use, regular passive smoking exposure, or unusually high consumption of home-grilled or charcoal-cooked foods in the 48 h prior to biological sampling. Demographic and anthropometric data were collected using structured questionnaires and standardized physical measurements [17].

### 2.2. Ethics Approval and Consent to Participate

Ethical approval for this study was obtained from the Human Experimentation Committee (HEC No. 39/67), Research Institute for Health Sciences (RIHES), Chiang Mai University, Chiang Mai, Thailand. All participants provided written informed consent prior to participation. The study was conducted in accordance with the Declaration of Helsinki.

### 2.3. Sample Collection

All participants provided urine and blood samples. The urine samples were collected in sterile containers and stored at −20 °C until analysis. Blood samples were drawn into EDTA or heparin tubes, which were then centrifuged at 3000× *g* for 10 min at a temperature of 4 °C within half an hour of collection [8]. Plasma was separated and preserved at either −20 °C or −80 °C until analysis, thereby preventing repeated freeze–thaw cycles [18].

All biological samples were collected post-shift at the end of the working week (Friday evening for workers) to capture cumulative occupational PAH exposure. Controls were sampled at matching times during their standard work schedule. Prior to blood pressure and heart rate measurements, all participants were required to sit quietly and rest for a minimum of 15 min in a quiet, temperature-controlled environment (24 °C ± 1 °C), Blood pressure and heart rate were measured using a standardized automated sphygmomanometer (Omron Healthcare Co., Ltd., Kyoto, Japan).

### 2.4. Determination of Urinary 1-Hydroxypyrene (1-OHP)

Urinary 1-hydroxypyrene (1-OHP) was used as a biomarker of internal exposure to polycyclic aromatic hydrocarbons (PAHs) [21]. Urine samples were analyzed using HPLC with fluorescence detection (HPLC-FLD; Agilent Technologies, Santa Clara, CA, USA) on a C18 reversed-phase column (4.6 × 250 mm, 5 µm; Agilent Technologies, Santa Clara, CA, USA) at 40 °C, with an isocratic mobile phase of acetonitrile (RCI Labscan Ltd., Bangkok, Thailand)/water (75:25, *v*/*v*) at 1.0 mL/min, with detection at λex = 242 nm and λem = 388 nm. Briefly, urine samples were enzymatically hydrolyzed using β-glucuronidase/arylsulfatase (Sigma-Aldrich, St. Louis, MO, USA) to release conjugated metabolites, followed by solid-phase extraction (SPE) using C18 SPE cartridges (Agilent Technologies, Santa Clara, CA, USA) for sample purification. The extracted analytes were then quantified using chromatographic analysis with external calibration curves. Validated analytical metrics included a linear calibration range of 0.005–2.0 µg/L, a limit of detection (LOD) of 0.002 µg/L, a limit of quantification (LOQ) of 0.006 µg/L, a recovery rate of 92.4%, and intra-day and inter-day coefficients of variation (CV%) of 2.74% and 7.20%, respectively. Quality control samples at low, medium, and high concentration levels were analyzed in each analytical batch to ensure method accuracy and reproducibility.

### 2.5. Creatinine Measurement and Normalization

The urinary creatinine concentrations were determined using a colorimetric method based on the Jaffé reaction with creatinine assay reagents (Human Gesellschaft für Biochemica und Diagnostica mbH, Wiesbaden, Germany). Creatinine levels were used to normalize urinary 1-OHP concentrations to account for urine dilution. Results were expressed as µg 1-OHP per g creatinine [21].

### 2.6. Measurement of Oxidative Stress and Biological Effect Biomarkers

Biomarkers of oxidative stress, lung epithelial injury, and DNA damage, including 8-hydroxy-2′-deoxyguanosine (8-OHdG), F_2_-isoprostanes (8-iso-PGF_2_α), Clara cell protein (CC16), and BPDE–DNA adducts, were measured using commercially available enzyme-linked immunosorbent assay (ELISA) kits (Guangzhou AORUIDA Biotechnology Co., Ltd., Guangzhou, China) according to the manufacturer’s instructions [12,18,19]. The manufacturer-validated performance characteristics were as follows: detection ranges of 1.56–100 ng/mL (8-OHdG), 15.63–1000 pg/mL (8-iso-PGF_2_α), 0.78–50 ng/mL (CC16), and as defined by the BPDE–DNA standard curve; assay sensitivities of 1 pg/mL (8-OHdG), 1 pg/mL (8-iso-PGF_2_α), 1 ng/mL (CC16), and 1 pg/mL (BPDE–DNA adducts). Intra-assay and inter-assay coefficients of variation (CV%) were less than 10% and less than 12%, respectively, for all four assays, consistent with manufacturer-reported specifications and accepted ELISA performance benchmarks. All samples were analyzed in duplicate, and laboratory operators were completely blinded to participant group codes throughout the analysis.

### 2.7. Statistical Analysis

Statistical analyses were performed using IBM SPSS Statistics for Windows, version 22.0 (IBM Corp., Armonk, NY, USA). The normality of continuous variables was assessed using the Shapiro–Wilk test. Since the data did not conform to a normal distribution, continuous variables are expressed as median (interquartile range, IQR; 25th–75th percentiles) and compared between groups using the Mann–Whitney U test. Categorical variables are presented as frequencies and percentages and compared using Fisher’s exact test. Correlations between biomarkers were evaluated using Spearman’s rank correlation coefficient (*r_s_*). Statistical significance was defined as *p* < 0.05 (two-tailed). For our comparison of *n* = 38 workers versus *n* = 20 controls, assuming 80% power (β = 0.20) and α = 0.05 (two-tailed), a post-hoc power analysis using G*Power, version 3.1 (Heinrich Heine University Düsseldorf, Düsseldorf, Germany), indicated that the minimum detectable effect size (MDES) for the Mann–Whitney U test is approximately *d* = 0.78, corresponding to a large effect size by Cohen’s conventions [22,23], indicating an elevated risk of Type II error for subtle or moderate molecular differences. The Hodges–Lehmann median ratio and its 95% confidence interval, estimated by bootstrap resampling (10,000 iterations), were calculated for statistically significant between-group comparisons as measures of effect size. Multiple linear regression models were constructed to evaluate the independent association between occupational group assignment and two primary outcomes—log-transformed urinary 1-OHP and resting heart rate—after simultaneous adjustment for potential confounders including age, BMI, and smoking status. Standardized regression coefficients (β), unstandardized estimates (B), and 95% confidence intervals are reported for each predictor. Model fit was assessed using the adjusted R^2^ and overall F-statistic.

## 3. Results

### 3.1. Demographic and Occupational Characteristics

The study groups were well-matched regarding age, BMI, and smoking status (*p* > 0.05). The median duration of employment for the worker group was 5 years, with 84.2% of workers working more than 6 h per day, as shown in Table 1.

### 3.2. Exposure, Physiological, and Biological Effect Markers

**Figure 1:**
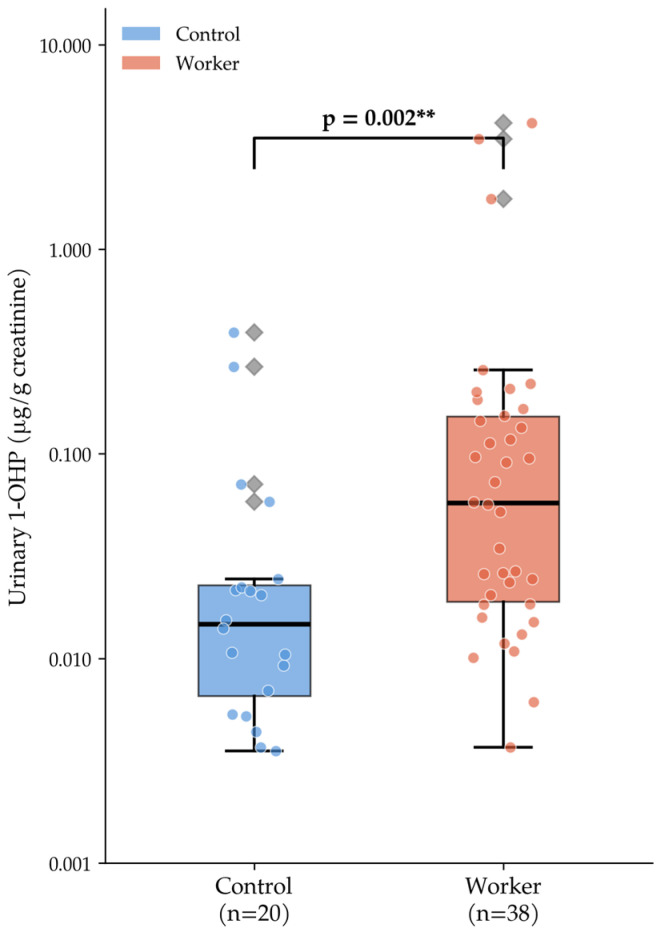
Box-and-whisker plot of urinary 1-hydroxypyrene (1-OHP) concentrations (µg/g creatinine) in the control group (*n* = 20) and grill restaurant worker group (*n* = 38). The *y*-axis is presented on a logarithmic scale. The box represents the interquartile range (IQR; 25th–75th percentiles), the horizontal line within the box indicates the median, whiskers extend to the most extreme non-outlier values, diamonds indicate outliers, and individual data points are shown as jittered dots. Statistical significance was assessed using the Mann–Whitney U test (** *p* = 0.002).

The results presented in Table 2 provide a detailed comparison of internal exposure, physiological responses, and biological effect markers between grill restaurant workers and the control group, offering important insights into the potential health impacts of occupational PAH exposure. The primary biomarker of internal exposure, urinary 1-hydroxypyrene (1-OHP), was markedly elevated among workers, with a median concentration approximately four times higher than that observed in the control group. Additionally, the interquartile range among workers was notably wider, indicating greater variability in exposure levels within this group. This finding clearly indicates substantial internal absorption of PAHs among grill restaurant workers and reflects ongoing occupational exposure in this environment. The distribution of urinary 1-OHP concentrations between groups is illustrated in Figure 1. The box-and-whisker plot confirms the markedly higher median exposure among workers compared to controls, with a wider interquartile range reflecting greater individual variability in occupational PAH absorption (*p* = 0.002).

**Figure 2:**
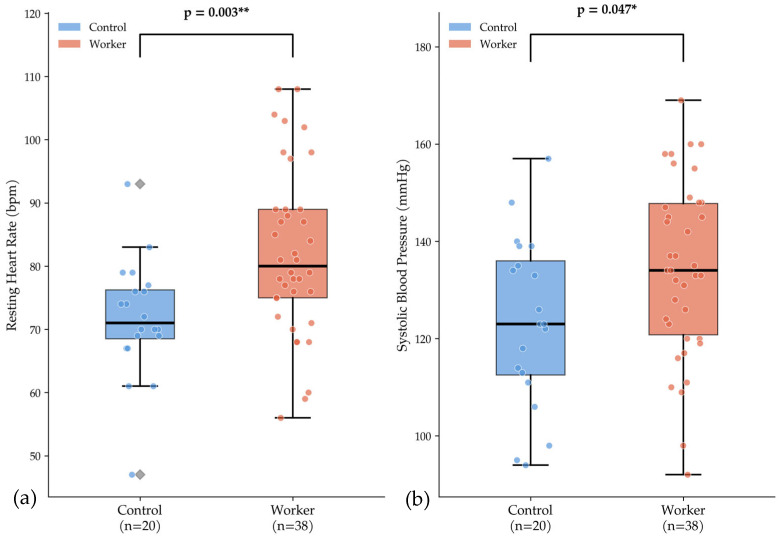
Box-and-whisker plots of cardiovascular parameters in the control group (*n* = 20) and grill restaurant worker group (*n* = 38); (**a**) resting heart rate (bpm) and (**b**) systolic blood pressure (mmHg). The box represents the IQR (25th–75th percentiles), the horizontal line indicates the median, whiskers extend to the most extreme non-outlier values, diamonds indicate outliers, and individual data points are shown as jittered dots. Statistical significance was assessed using the Mann–Whitney U test (** *p* = 0.003 for heart rate; * *p* = 0.047 for systolic blood pressure).

With respect to physiological parameters, the data provide significant evidence of cardiovascular strain among exposed workers. Both systolic blood pressure and resting heart rate were significantly elevated compared with controls, highlighting a potential acute stress response associated with occupational factors such as heat exposure, physical workload, and inhalation of combustion-related pollutants. The elevation in heart rate was particularly pronounced, with workers exhibiting both higher median values and a broader distribution, which may indicate sustained sympathetic activation. Although diastolic blood pressure was also higher in the worker group, the difference did not reach statistical significance, suggesting that systolic pressure and heart rate may serve as more sensitive indicators of early physiological stress in this context. Figure 2 illustrates the distributions of resting heart rate and systolic blood pressure between groups. Workers exhibited both higher median values and broader distributions for both parameters, consistent with sustained cardiovascular strain. Resting heart rate was significantly elevated among workers (*p* = 0.003), as was systolic blood pressure (*p* = 0.047), while individual data points and outliers were visible, confirming that these findings are not driven by a small number of extreme values.

In contrast, biomarkers of biological effects, including oxidative stress, lung epithelial injury, and genotoxicity, did not differ significantly between the two groups. Specifically, levels of oxidative stress markers such as 8-OHdG and 8-iso-PGF_2_α were nearly identical, indicating no detectable increase in systemic oxidative damage despite elevated exposure. Similarly, the lung injury marker CC16 showed no meaningful elevation among workers, suggesting that epithelial damage in the respiratory tract may not yet be apparent. Furthermore, concentrations of BPDE-DNA adduct, which serve as indicators of PAH-induced genotoxicity, were comparable between groups, implying that DNA damage has not accumulated to a measurable extent under the current exposure conditions. Non-significant findings for biological effect biomarkers are discussed in the context of statistical power in Section 4.2.

Taken together, these findings suggest a dissociation between exposure and downstream biological effects. While grill restaurant workers experience significantly higher internal PAH exposure and exhibit clear signs of acute physiological strain, these exposures have not yet resulted in measurable oxidative stress, lung injury, or DNA damage. This may reflect relatively low-to-moderate exposure levels, effective physiological adaptation, or an insufficient duration of exposure to elicit detectable biological damage. Importantly, the results highlight that cardiovascular parameters, particularly heart rate and systolic blood pressure, may function as more sensitive early indicators of occupational health risk in this population than traditional biomarkers of oxidative stress or genotoxicity.

### 3.3. Correlation Patterns Among Biomarkers

The Spearman correlation analysis examining the relationships between the exposure biomarker, urinary 1-hydroxypyrene (1-OHP), and multiple biological effect markers, including 8-iso-PGF1α, CC16, BPDE-DNA adducts, and 8-OHdG, across the entire study population (*n* = 58) was presented in Table 3. The findings reveal two clear patterns in the correlation structure [19].

First, the association between PAH exposure and biological effects appears weak and non-significant [18]. Urinary 1-OHP demonstrated only minimal correlations with all evaluated biomarkers, with coefficients ranging from −0.031 for 8-OHdG to 0.125 for CC16 [17]. None of these relationships reached statistical significance, indicating that the internal dose of PAHs, as reflected by 1-OHP, does not show a clear or direct linear relationship with markers of oxidative stress, lung injury, or genotoxicity in this cohort [16,24]. This may suggest that the exposure levels are below a threshold required to elicit measurable systemic biological responses, or that individual variability, exposure duration, or adaptive mechanisms may obscure a straightforward exposure–response relationship [9,19].

In contrast, strong and statistically significant intercorrelations were observed among the biological effect markers themselves, highlighting a coordinated physiological response [7,20]. The most prominent association was between 8-iso-PGF1α, a marker of lipid peroxidation, and BPDE-DNA adducts, with a strong positive correlation (*r_s_* = 0.628, *p* < 0.01) [18]. Similarly, 8-iso-PGF1α was strongly correlated with 8-OHdG (*r_s_* = 0.597, *p* < 0.01), indicating a close link between lipid peroxidation and oxidative DNA damage [12,19]. Furthermore, BPDE-DNA adducts and 8-OHdG were also significantly correlated (*r_s_* = 0.547, *p* < 0.01), suggesting that different pathways of DNA damage—direct adduct formation and oxidative modification—may occur concurrently or share common underlying mechanisms [21,22].

Additionally, CC16, a biomarker of lung epithelial integrity, showed moderate but significant correlations with all other biological markers, including 8-iso-PGF1α (*r_s_* = 0.433), BPDE-DNA adducts (*r_s_* = 0.433), and 8-OHdG (*r_s_* = 0.424) [3]. These findings imply that pulmonary epithelial responses are linked with systemic oxidative stress and genotoxic processes, further supporting the concept of an integrated biological response to environmental or occupational stressors [7].

Overall, the correlation patterns suggest that there is a dissociation between exposure and measurable biological effects, alongside a strong internal coherence among the biological response markers [24]. Although urinary 1-OHP does not appear to directly predict downstream biological damage in a linear manner, the significant interrelationships among oxidative stress, DNA damage, and lung injury markers indicate that these endpoints are biologically interconnected and may reflect a unified response pathway [7,20]. This reinforces the notion that once biological effects are initiated, they tend to progress in a coordinated way even in the absence of a strong observable correlation with the measured exposure biomarker [9].

### 3.4. Multivariate Regression Analysis

Multiple linear regression models were constructed to examine whether occupational group remained a significant predictor of urinary 1-OHP and resting heart rate after adjusting for potential confounders including age, BMI, and smoking status (Table 4).

For urinary 1-OHP, the occupational group was a significant independent predictor (β = 0.366, *p* = 0.005), indicating that grill workers had substantially higher internal PAH exposure compared to controls, independent of age, BMI, and smoking status. The overall model explained 12.6% of the variance in urinary 1-OHP (adjusted R^2^ = 0.126, *F*(4,53) = 3.050, *p* = 0.025). None of the covariates reached statistical significance, suggesting that the observed elevation in PAH exposure was attributable primarily to occupational exposure rather than individual characteristics.

Similarly, for resting heart rate, occupational group remained a significant independent predictor (β = 0.374, *p* = 0.004), confirming that grill workers exhibited higher resting heart rates independent of age, BMI, and smoking status. The overall model accounted for 11.2% of the variance in resting heart rate (adjusted R^2^ = 0.112, *F*(4,53) = 2.803, *p* = 0.035). Age, BMI, and smoking status were not significant covariates, indicating that the elevated heart rate observed among workers was not explained by demographic or lifestyle factors alone.

Taken together, these results confirm that occupational worker status is a significant independent predictor of both elevated urinary 1-OHP and accelerated resting heart rate, reinforcing the conclusion that grill restaurant work poses a distinct physiological burden beyond that attributable to individual confounders.

## 4. Discussion

The present study employed an integrated biomonitoring approach to evaluate occupational exposure to polycyclic aromatic hydrocarbons (PAHs) and its associated biological effects among grill restaurant workers [3,7]. Our findings demonstrate a clear selective association between high internal exposure doses and systemic biological damage, while highlighting a significant, often overlooked, physiological strain on the cardiovascular system [25,26].

### 4.1. Occupational PAH Exposure and Internal Dose

The results confirmed that grill restaurant workers face significantly higher occupational exposure to PAHs than the control group, with median urinary 1-OHP levels approximately 3.9-fold higher (0.057 vs. 0.015 µg/g creatinine; HL ratio = 3.66, 95% CI: 1.68–7.12) [16,17]. This is consistent with the high-temperature grilling process, where meat fats and juices drip onto charcoal or hot surfaces, undergoing incomplete combustion and thermal degradation to release concentrated PAH-laden smoke [14,15].

An observational pattern identified in our results suggests a potential exposure gradient based on self-reported smoke proximity [4]. Workers who reported being ‘always’ near smoke sources exhibited 1-OHP levels roughly ten times higher than the control baseline [17], though this association should be interpreted cautiously as a self-reported observational finding rather than a confirmed exposure gradient, given the absence of direct airborne PAH or PM_2.5_ measurements. Furthermore, the lack of significant difference in 1-OHP levels between workers in “ventilated” versus “non-ventilated” areas suggests that current general ventilation systems in these establishments are inadequate for capturing fine particulate matter and gaseous PAHs at the source [11,16].

### 4.2. Limited Association Between PAH Exposure and Genotoxicity Markers

Despite the elevated internal dose of PAHs, this study did not find statistically significant differences in biomarkers of oxidative stress (8-OHdG, F_2_-isoprostanes), lung epithelial injury (CC16), or genotoxicity (BPDE-DNA adducts) between workers and controls [17,24]. Several factors may explain this “latency” in biological response [19]:

• Threshold Effect: The exposure levels, while significantly higher than controls, may still fall below the threshold required to overwhelm cellular antioxidant defenses and DNA repair mechanisms in the short term [9,18].
• Transient Nature of Biomarkers: Oxidative stress markers are highly dynamic and influenced by recent lifestyle factors, metabolic capacity, and the specific timing of sample collection relative to the work shift [12,16].
• Complexity of Metabolism: The formation of BPDE-DNA adducts is a complex multi-step process [19,20]. The absence of significant differences does not necessarily indicate a lack of risk but rather reflects the intricate exposure-response relationship where individual genetic susceptibility and DNA repair efficiency play major roles [19].

It should be noted, however, that the absence of statistical significance should not be interpreted as evidence of the absence of a biological effect. Given the minimum detectable effect size (MDES) of *d* = 0.78 for this sample configuration, subtle or moderate molecular differences may not have been detectable with the current sample size, and the possibility of Type II error cannot be excluded.

### 4.3. Cardiovascular Strain and Occupational Co-Exposures

Perhaps the most notable finding is the significant elevation in systolic blood pressure (*p =* 0.047) and resting heart rate (*p =* 0.003) among workers [27,28]. Although DNA damage remains undetectable, the cardiovascular system appears to be under acute stress [25]. We attribute this to the combined influence of chemical inhalation and plausible unmeasured occupational co-exposures, including ambient heat and physical workload, which were not directly quantified in the present study [27,29].

Kitchen environments in grill restaurants involve proximity to open charcoal fires and high ambient temperatures [11,27]. This environment triggers thermoregulatory strain, increasing sympathetic nervous system activity and cardiac output [26,28]. When combined with the inhalation of PAHs and fine particulate matter, these factors can trigger autonomic imbalance and systemic inflammation [25,30]. Similar to the findings for foundry workers and firefighters, the “dual-threat” of heat and smoke in the kitchens poses a cumulative cardiovascular risk [25,26]. The higher median heart rate in our worker cohort (80.0 bpm vs. 71.0 bpm in controls) serves as a sensitive indicator of acute occupational physiological response, consistent with differential biomarker sensitivity and kinetics rather than a temporal sequence of events [28,31].

It should be noted, however, that the elevated cardiovascular parameters observed among workers cannot be attributed exclusively to PAH inhalation, as heat exposure, physical workload, hydration status, and other occupational stressors were not directly quantified in this study. These unmeasured factors may independently or jointly contribute to the observed elevations in resting heart rate and systolic blood pressure, and their potential contributions cannot be excluded [26,27,29].

PAH-induced ROS generation is well documented in the literature; PAHs undergo metabolic activation via cytochrome P450 enzymes to form ROS, which can initiate a cascade of oxidative damage to cellular lipids, proteins, and nucleic acids [32]. However, the magnitude of the oxidative stress response is highly dose-dependent, and studies have reported that oxidative stress biomarker levels were higher in workers with high PAH exposure than in those with mild exposure, although differences were not always statistically significant at lower exposure tiers [33]. The relatively low-to-moderate internal PAH exposure levels in this cohort, as reflected by urinary 1-OHP values, may not have been sufficient to overwhelm endogenous antioxidant defense systems to produce measurable elevations in 8-OHdG or 8-iso-PGF_2_α. Furthermore, dietary habits may act as a powerful modulating factor. The traditional Northern Thai diet contains a high density of fresh herbs, spices, and phytochemicals—including phenolics and flavonoids—with well-documented free radical scavenging and antioxidant capacities [34,35]. It is plausible that this dietary background provided sufficient antioxidant buffering capacity to attenuate systemic lipid peroxidation and oxidative DNA damage, keeping 8-OHdG and 8-iso-PGF_2_α levels stable despite elevated internal PAH exposure. These explanations, however, remain speculative in the absence of direct measurements of dietary antioxidant status, individual metabolic capacity, or adaptive physiological markers. They are therefore proposed as testable hypotheses for future longitudinal investigation rather than conclusions drawn from the present data.

### 4.4. Occupational Health and Policy Implications

From a public health perspective, these findings shift the focus from long-term carcinogenesis to immediate physiological welfare [25]. The prevalence of chronic cough and respiratory symptoms among workers, together with the cardiovascular strain, underlines the need for a multifaceted intervention strategy [11,28]:

(1) Engineering Controls: Transitioning from general ventilation to Local Exhaust Ventilation (LEV) installed directly above grilling surfaces is essential to reduce the breathing-zone concentration of PAHs [11,28].
(2) Health Surveillance: Occupational health protocols for the food service sector should expand beyond respiratory checks to include regular blood pressure and heart rate monitoring [25,31].
(3) Administrative Controls: Managing plausible heat-related occupational co-exposures through mandatory hydration protocols, cooling stations, and task rotation is vital to reducing cumulative cardiovascular burden [25,36].

### 4.5. Strengths and Limitations

The strengths of this study are in its simultaneous evaluation of exposure, biological effect markers, and physiological vital signs, providing a holistic view of the worker’s health status [3,7]. However, the cross-sectional design prevents establishing a definitive causal timeline, and the small sample size may limit the ability to detect subtle molecular changes [17]. Future research should utilize longitudinal designs and incorporate direct environmental air monitoring to complement biomonitoring data [3,4].

Additionally, formal matching for socioeconomic status, dietary patterns beyond the 48 h pre-sampling exclusion protocol, and quantified traffic-related PAH exposure was not performed, which may introduce residual confounding that cannot be fully excluded. Airborne PAH concentrations, PM_2.5_ levels, benzo[a]pyrene, and WBGT heat-index measurements were not collected in the workplace. The smoke proximity gradient reported in this study was based on worker-reported questionnaire data rather than instrumented air monitoring, and exposure gradient conclusions are therefore limited to internal dose comparisons via urinary 1-OHP. Intra-individual variability in oxidative stress markers such as 8-OHdG and 8-iso-PGF_2_α, which can fluctuate with dietary antioxidant intake, physical exertion, and diurnal rhythms, was not formally controlled beyond the 48 h pre-sampling dietary exclusion protocol. The post-hoc power analysis indicated an MDES of *d* = 0.78, confirming that the study was adequately powered to detect only large effect sizes. Subtle or moderate differences in oxidative stress and genotoxicity biomarkers may therefore have remained undetected, and the non-significant findings for these endpoints should be interpreted with appropriate caution.

Furthermore, healthy-worker bias may have influenced our findings, as workers who remain employed in grill restaurants may represent a self-selected, healthier subset of the exposed population, potentially underestimating the true burden of occupational PAH exposure. Furthermore, no correction for multiple statistical comparisons was applied in the present study, which increases the risk of Type I error and should be considered when interpreting the reported *p*-values.

## 5. Conclusions

This study concludes that grill restaurant workers in Northern Thailand sustain significantly elevated internal PAH exposure, as quantified by urinary 1-OHP levels approximately four times higher than those of office-based controls. Importantly, this internal dose elevation is accompanied by significant cardiovascular strain—reflected by elevated systolic blood pressure and resting heart rate—that remained significant after full adjustment for age, BMI, and smoking status (β = 0.366, *p* = 0.005 and β = 0.374, *p* = 0.004, respectively), confirming that occupational exposure is the primary driver of these physiological changes.

The absence of significant group differences in oxidative stress (8-OHdG, 8-iso-PGF_2_α), lung epithelial injury (CC16), and genotoxicity (BPDE–DNA adducts) should be interpreted as an important negative result rather than evidence of biological safety. Given the minimum detectable effect size of *d* = 0.78 for this sample configuration, subtle molecular alterations may remain undetected, and longitudinal studies with larger cohorts are needed to evaluate cumulative biological harm over extended occupational exposure timelines.

These findings collectively conclude that cardiovascular parameters—particularly resting heart rate and systolic blood pressure—serve as sensitive early indicators of occupational health risk in grill restaurant workers, detectable prior to measurable genomic damage. Current general ventilation systems appear inadequate, and localized engineering controls, routine cardiovascular health monitoring, and provision of personal protective equipment are warranted as priority public health interventions in this underrepresented occupational sector.

## Figures and Tables

**Table 1:** Demographic, anthropometric, and occupational characteristics of grill restaurant workers and healthy controls.

Characteristics	Median (IQR)		*p*-Value
	Control Group (*n* = 20)	Worker Group (*n* = 38)	
Age (years)	37.0 (27.8–45.8)	38.5 (29.8–48.5)	0.684
Height (cm)	163.5 (155.8–171.0)	160.0 (155.0–166.8)	0.441
Weight (kg)	64.0 (53.8–78.8)	63.3 (55.0–74.5)	0.985
BMI (kg/m^2^)	24.8 (22.0–26.5)	24.7 (21.9–27.8)	0.941
Work Duration (years)	—	5.0 (2.0–10.0)	—
Daily Work Hours			
<6 h	—	6 (15.8%)	—
>6 h	—	32 (84.2%)	—
Smoking Status			
Non-smoker	18 (90.0%)	33 (86.8%)	1.000
Smoker	2 (10.0%)	5 (13.2%)	

Continuous values are presented as median (interquartile range, IQR; 25th–75th percentiles). Categorical variables are presented as frequency (percentage). *p*-values were calculated using the Mann–Whitney U test for continuous variables and Fisher’s exact test for categorical variables.

**Table 2:** Comparison of internal PAH exposure biomarkers, physiological vital signs, and downstream biological effect markers between study groups.

Parameters	Median (IQR)		*p*-Value	HL Ratio (95% CI)
	Control Group (*n* = 20)	Worker Group (*n* = 38)		
Exposure Biomarker				
Urinary 1-OHP (µg/g creatinine)	0.014 (0.007–0.032)	0.057 (0.032–0.141)	0.002 **	3.66 (1.68–7.12)
Physiological Markers				
Systolic BP (mmHg)	123.0 (112.5–136.0)	134.0 (120.8–147.8)	0.047 *	1.09 (1.00–1.18)
Diastolic BP (mmHg)	75.5 (70.8–82.0)	82.5 (73.0–88.8)	0.128	—
Heart rate (bpm)	71.0 (68.5–76.2)	80.0 (75.0–89.0)	0.003 **	1.13 (1.05–1.23)
Biological Effect Markers				
8-OHdG (ng/mL)	70.9 (64.3–83.5)	71.1 (63.2–104.2)	0.854	—
8-iso-PGFalpha (pg/mL)	24.4 (20.3–30.6)	23.3 (20.1–26.2)	0.412	—
CC16 (ng/mL)	17.7 (14.2–22.6)	18.4 (15.5–24.1)	0.435	—
BPDE-DNA adduct (pg/mL)	24.8 (18.1–28.9)	24.3 (19.4–33.4)	0.621	—

Continuous values are presented as median (interquartile range, IQR; 25th–75th percentiles). Categorical variables are presented as frequency (percentage). *p*-values were calculated using the Mann–Whitney U test for continuous variables and Fisher’s exact test for categorical variables. HL ratio = Hodges–Lehmann median ratio (Worker/Control); 95% CI estimated by bootstrap (10,000 iterations); — indicates non-significant comparisons for which effect estimation is not reported. Significance levels are indicated as follows: * *p* < 0.05 and ** *p* < 0.01.

**Table 3:** Spearman Correlation Matrix Between Biomarkers.

Biomarkers	1-OHP	8-iso-PGF1α	CC16	BPDE	8-OHdG
1-OHP	1				
8-iso-PGF1α	0.074	1			
CC16	0.125	0.433 **	1		
BPDE	0.065	0.628 **	0.433 **	1	
8-OHdG	−0.031	0.597 **	0.424 **	0.547 **	1

Values represent Spearman’s rank correlation coefficients (*r_s_*). A double asterisk indicates statistical significance at *p* < 0.01 **.

**Table 4:** Multiple Linear Regression Analysis of Occupational Group on Urinary 1-OHP and Resting Heart Rate, Adjusted for Age, BMI, and Smoking Status.

Variable	β	95% CI	*p*-Value
Outcome: log (Urinary 1-OHP)			
Occupational group (worker)	0.366	0.391–2.074	0.005
Age	−0.186	−0.093–0.014	0.141
BMI	0.067	−0.050–0.086	0.593
Smoking status	0.066	−2.252–3.885	0.596
Adjusted R^2^ = 0.126, *F*(4,53) = 3.050, *p* = 0.025			
Outcome: Heart Rate (bpm)			
Occupational group (worker)	0.374	3.329–17.013	0.004
Age	−0.113	−0.628–0.239	0.372
BMI	0.111	−0.307–0.796	0.378
Smoking status	0.052	−19.774–30.132	0.679
Adjusted R^2^ = 0.112, *F*(4,53) = 2.803, *p* = 0.035			

β = standardized regression coefficient; CI = confidence interval. Occupational group coded as 0 = control, 1 = worker. Smoking status coded as 0 = non-smoker, 1 = smoker.

## Data Availability

The original contributions presented in this study are included in the article. Further inquiries can be directed to the corresponding author.
